# Real-time observations of lithium battery reactions—operando neutron diffraction analysis during practical operation

**DOI:** 10.1038/srep28843

**Published:** 2016-06-30

**Authors:** Sou Taminato, Masao Yonemura, Shinya Shiotani, Takashi Kamiyama, Shuki Torii, Miki Nagao, Yoshihisa Ishikawa, Kazuhiro Mori, Toshiharu Fukunaga, Yohei Onodera, Takahiro Naka, Makoto Morishima, Yoshio Ukyo, Dyah Sulistyanintyas Adipranoto, Hajime Arai, Yoshiharu Uchimoto, Zempachi Ogumi, Kota Suzuki, Masaaki Hirayama, Ryoji Kanno

**Affiliations:** 1 Department of Chemical Science and Engineering, School of Materials and Chemical Technology, Tokyo Institute of Technology, 4259 Nagatsuta-cho, Midori-ku, Yokohama 226-8502, Japan; 2 Institute of Materials Structure Science, High Energy Accelerator Research Organization, 1-1 Oho, Tsukuba, Ibaraki 305-0801, Japan; 3 Sokendai (The Graduate University for Advanced Studies), Shirakata 203-1, Tokai, Naka 319-1106, Japan; 4 Office of Society-Academia Collaboration for Innovation, Kyoto University, Uji-shi, Kyoto 611-0011, Japan; 5 Research Reactor Institute, Kyoto University, Kumatori-cho, Sennan-gun, Osaka 590-0494, Japan; 6 Graduate School of Human and Environmental Studies, Kyoto University, Sakyo-ku, Kyoto 606-8501, Japan

## Abstract

Among the energy storage devices for applications in electric vehicles and stationary uses, lithium batteries typically deliver high performance. However, there is still a missing link between the engineering developments for large-scale batteries and the fundamental science of each battery component. Elucidating reaction mechanisms under practical operation is crucial for future battery technology. Here, we report an operando diffraction technique that uses high-intensity neutrons to detect reactions in non-equilibrium states driven by high-current operation in commercial 18650 cells. The experimental system comprising a time-of-flight diffractometer with automated Rietveld analysis was developed to collect and analyse diffraction data produced by sequential charge and discharge processes. Furthermore, observations under high current drain revealed inhomogeneous reactions, a structural relaxation after discharge, and a shift in the lithium concentration ranges with cycling in the electrode matrix. The technique provides valuable information required for the development of advanced batteries.

Since the commercialization of secondary lithium batteries in 1991^1^, this excellent system of electrochemical energy storage has been assiduously developed, and its uses have expanded from small batteries to a much wider range of large-scale applications^2^,^3^ in which high reliability, safety, and long-term stability are required^4^. To improve these characteristics, battery reactions under practical usage conditions must be better understood^5^.

In commercial batteries, the most common configuration is the 18650 cylindrical cell, with an 18 mm diameter and 65 mm height; these are used for laptop computers and even for electric vehicle (EV) applications. Reactions in these practical batteries proceed in a non-equilibrium state within an electrode matrix composed of conducting agents, adhesive additives, and electrode materials. In addition, the extremely high current caused by the pulsed operational conditions, for example, makes the battery reactions non-equilibrium and non-homogeneous; this may induce a lithium concentration gradient in the electrode matrix which will lead to relaxation processes after the current passes through the cell^6^,^7^,^8^. Non-equilibrium conditions may also change the lithium concentration ranges used for the reactions in the electrodes, which is a key factor for balancing the lithium between the cathode and anode, and thus, for designing batteries^8^,^9^. The development of experimental tools to detect and analyse these reactions under practical conditions is urgently necessary for improved battery design.

There are several *in situ* and operando analytical methods that can be used to clarify the reaction mechanism for electrode materials during full cell operation, including energy-dispersive X-ray diffraction (EDXRD)^10^, energy-scanning confocal X-ray diffraction (ES-XRD)^11^, nuclear magnetic resonance (NMR)^12^, and small-angle neutron scattering^13^. Among the *in situ* and operando techniques proposed for elucidating the reactions, a technique that allows structural analysis would provide essential and fundamental information^14^. Operando measurement using high-intensity and high-resolution diffraction is one of the best techniques for observing the changes in the cell components during operation, which may provide clues that help us to recognize the significant issues affecting the deterioration of the battery. However, operando systems are rather difficult to apply in commercial-size cells with stainless-steel cans. Many approaches have therefore been proposed based on stationary neutrons^15^ and high-energy X-rays^16^. In the neutron techniques, structural changes in the electrode materials have been detected under conditions for slow reaction kinetics or with a repeated data accumulation process. However, comprehensive observations that can detect deterioration-related changes in the components under actual operation remain difficult.

We developed an experimental system that uses pulsed neutrons and a new time-of-flight (TOF)-type diffractometer with high-resolution^17^ and high-intensity^18^, and enables operando measurements in large commercial cells under high current drain conditions. The structural changes in the cathode and anode materials were simultaneously and directly analysed at each charge-discharge reaction step, and the relaxation process was detected under non-equilibrium battery-reaction conditions. The new measurement system could provide information for a wide range of reactions related to real battery operation conditions.

## Results

### Development of the experimental setup

#### Graphical analysis of the sequentially collected data

**Figure 1:**
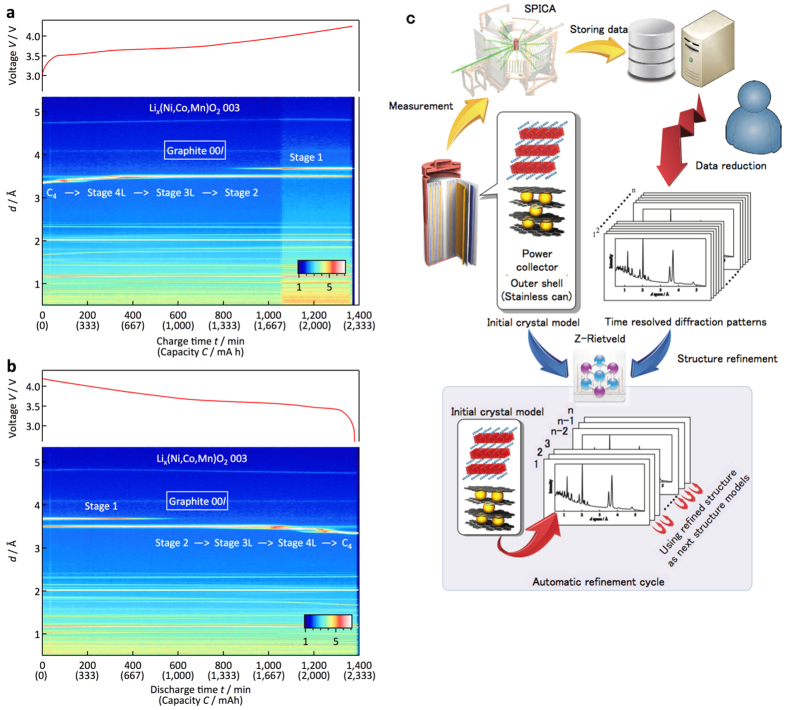
Typical diffraction profile output during battery operation and schematic of refinement system. Sample total diffraction profiles of a battery during charge (**a**) and discharge (**b**) experiments. The voltage profiles of the charge and discharge reactions are indicated above each diffraction profile. (**c**) Schematic diagram and cell environment for the *in situ* experiment using an 18650 cell in the SPICA diffractometer. Examples of the data analysis using the data analysis program developed in the present study. Schematic diagram of the flow chart for the analysis process is indicated for the automatic refinement cycle.

Figure 1a,b show examples of total diffraction profiles collected under various charge and discharge current conditions. All the data acquired in the present study are shown in Supplementary Fig. S1. Each diffraction profile contains all the diffraction peaks from the battery components, i.e. the cathode (lithium nickel manganese cobalt oxide: NMC), anode (C), current corrector (Al, Cu), and battery can (Fe). The graphical data in the diffraction profiles provide clear and simultaneous information about the structural changes occurring in both the cathode and anode materials. The structural changes of the carbon anode in the 18650 cell are consistent with those reported previously^9^,^19^,^20^,^21^,^22^: C (graphite) ↔ stage 4L (LiC_24_) ↔ stage 3L (LiC_18_) ↔ stage 2 (LiC_12_) ↔ stage 1 (LiC_6_). Precise structural changes under high-current conditions will be discussed later. Although no distinct changes in the graphical data were observed for the NMC cathode, variations in the lattice parameters determined by the Rietveld refinements indicate reversible phase changes during the cycle (see below)^23^.

#### Rietveld analysis of the sequentially collected data

**Figure 2:**
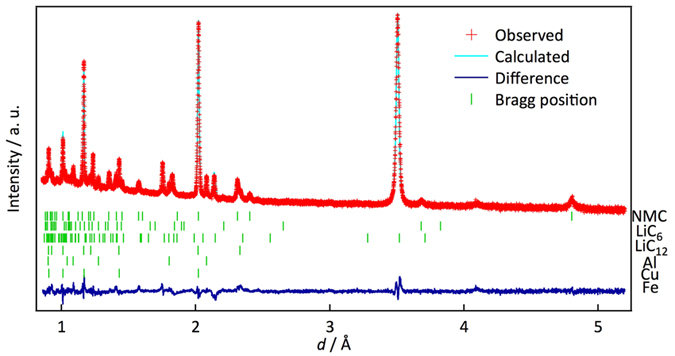
Rietveld-refined neutron diffraction pattern. Example of the Rietveld-refinement pattern after the automatic refinement process. Green dashes indicate the positions of Bragg reflections for the battery components.

**Figure 3:**
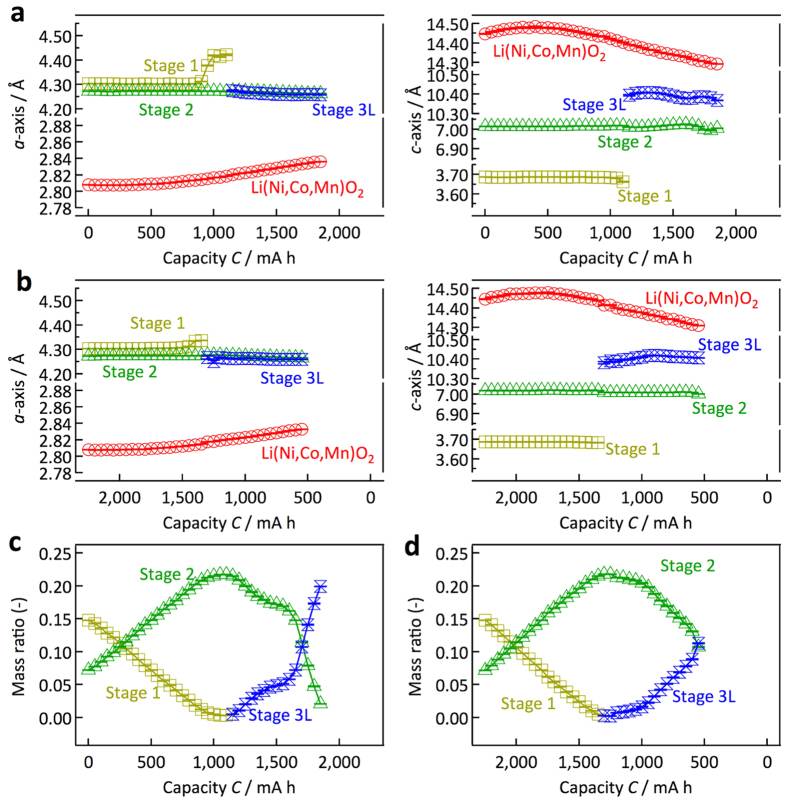
Lattice parameters and mass ratio changes refined by automatic analysis. Lattice parameter changes of the cathode and anode materials for discharge (**a**) and charge (**b**) processes at 0.05 C. The discharge reaction proceeds from the left- to the right-hand side, and the charge reaction proceeds from the right- to the left-hand side in the figures. The lattice parameters *a* and *c* are plotted as functions of the cell capacity. The cathode material (NMC) and the anode material (carbon) have hexagonal symmetry, with *a* and *c* as lattice parameters. Changes in the mass ratio of Li-C compounds at a C rate of 0.05 for the discharge (**c**) and charge (**d**) processes.

An automatic data analysis procedure is a prerequisite for handling the large number of diffraction data sets provided by the operando observation and sequential data accumulation routine at each charge and discharge process. Figure 1c schematically illustrates the data analysis procedure developed in the present study. Figure 2 shows an example of the refinement results. The initial parameters for the refinements in the multiple data analyses were obtained from previously reported data for a carbon anode^24^, NMC cathode^25^, Fe, Cu, and Al^26^,^27^,^28^. The good agreement factor *R*_wp_ and goodness of fit factor *S* (= *R*_wp_/*R*_e_, *R*_e_: minimum of *R*_wp_ statistically expected) were 3.39% and 1.78, respectively, which indicates that the automatic refinement provides reasonable structural parameters for the individual phases of the experimental diffraction data during battery operation. The lattice parameters, the ratio of the phases present in the cell, and the structural parameters of each phase were automatically refined by the automatic Rietveld refinement routine that we developed. It is worth emphasizing that our operando data collection at each charge or discharge step provides diffraction data that is sufficiently intense for Rietveld analysis. Figure 3 summarizes the lattice parameter changes of the cathode and anode together with the mass ratio of each Li–C compound that appears at a C rate of 0.05. The NMC cathode shows an increase in the *c* lattice parameter with a decrease in the *a* lattice parameter in the hexagonal *R-*3 m structure. In the deep charge region, the decrease in the *c* parameter might be accompanied by a phase change from a cubic close packing (ccp) structure to a hexagonal close packing (hcp) structure. For the anode, the lattice parameters and mass ratio changes of each phase were determined. The automatic analysis was quite successful for elucidating the reaction processes taking place in the battery can.

### Reactions observed for the 18650 cells under operando measurements

Our data accumulation setup elucidated the reaction mechanism at each charge and discharge step during battery operation. The observations include (i) the lithium concentration in the cell; (ii) the differences in the kinetics and the reaction mechanism between charge and discharge processes; (iii) the non-equilibrium reactions in the anode; and (iv) the non-equilibrium reactions in the cathode. The structural relaxation process of the electrodes in the commercial cell was clarified under high current drain for the first time.

#### Lithium concentration in the cell

The lithium-concentration balance between the cathode and anode during the cycles is a key factor for designing batteries because the change in concentration is strongly correlated with formation of the solid electrolyte interphase (SEI) and the deterioration of electrodes, which affects the long-term stability and reliability of the batteries^29^,^30^. As high-intensity data is required to determine the lithium concentration of the electrodes during battery operation, this type of observation was not previously possible for each charge and discharge process.

**Figure 4:**
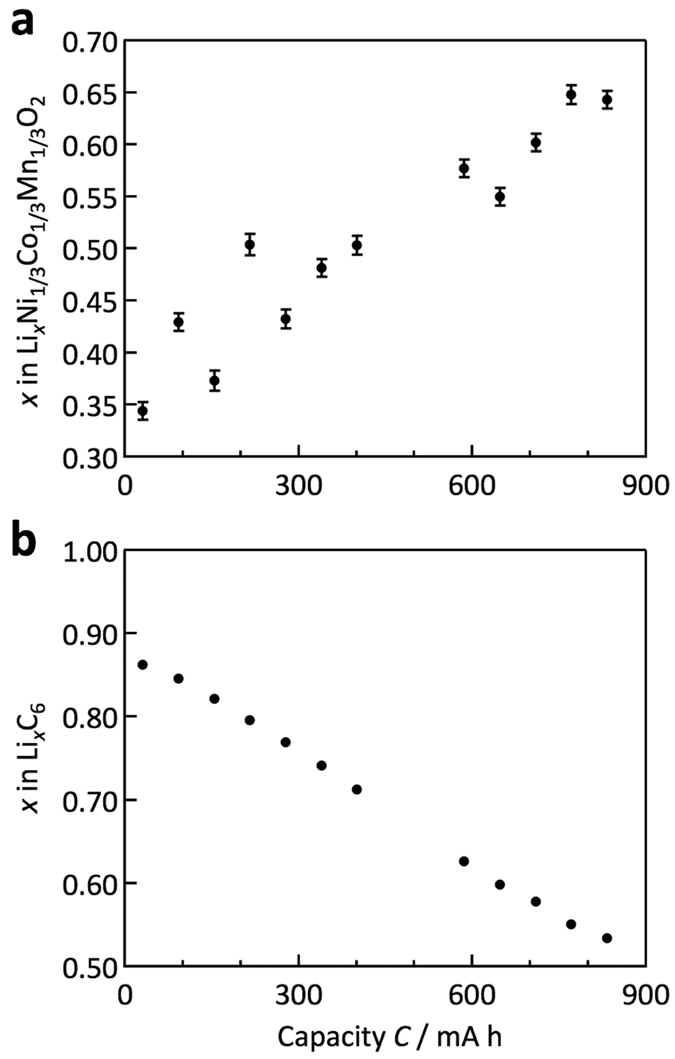
Lithium composition refined by Rietveld analysis. Lithium-composition changes for cathode (**a**) and anode (**b**) materials as a function of the capacity of the lithium cell.

The diffraction data was collected using the 0.1 C rate for quantitative lithium concentration analysis. The lithium compositions in the cathode were thus directly determined by the Rietveld analysis. For the anode, the stage 1 (LiC_6_)/stage 2 (LiC_12_) ratio was determined for the region where the stage 1 and stage 2 phases coexist. Supplementary Fig. S2 shows examples of the Rietveld refinement patterns in the initial and after-discharge states. Supplementary Table S1 summarizes the refinement results for these experiments. Figure 4 shows the composition changes of both the cathode and the anode as a function of the capacity of the lithium cell. Although the lithium content in the deep state of charge region for the cathode is slightly distributed, the analysis successfully indicates that the direct measurement of the lithium content is possible during battery operation. The composition regions *x* used for the reversible reactions were 0.5–0.87 and 0.35–0.65 for Li_*x*_C_6_ and Li_*x*_(Ni_1/3_Co_1/3_Mn_1/3_)O_2_, respectively, which were used for the 0–900 mAh region of the cell. This information provides basic battery reaction data to clarify the deterioration mechanism during cycling and under high-temperature operation^31^ and storage conditions^32^.

#### Changes in the reaction mechanism between the charge and discharge processes

**Figure 5:**
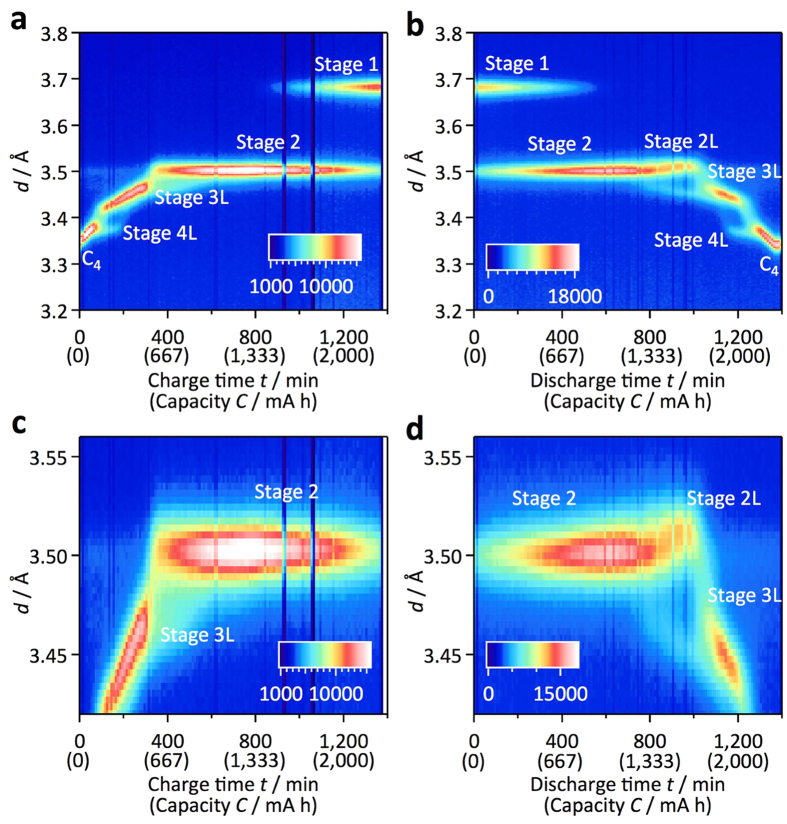
Different phase transition behaviour of graphite anode between the charge-discharge reactions. Two-dimensional profiles for the carbon 00*l* peak during the charge (**a**,**c**) and discharge (**b**,**d**) processes at the 0.05 C rate show the stage-related structural changes of the graphite anode. The profiles around the stage (2/3L) regions are shown in (**c**,**d**) for the charge and discharge processes, respectively. The phase transition from stage 2 to stage 3L through stage 2L is observed only for the discharge process.

**Figure 6:**
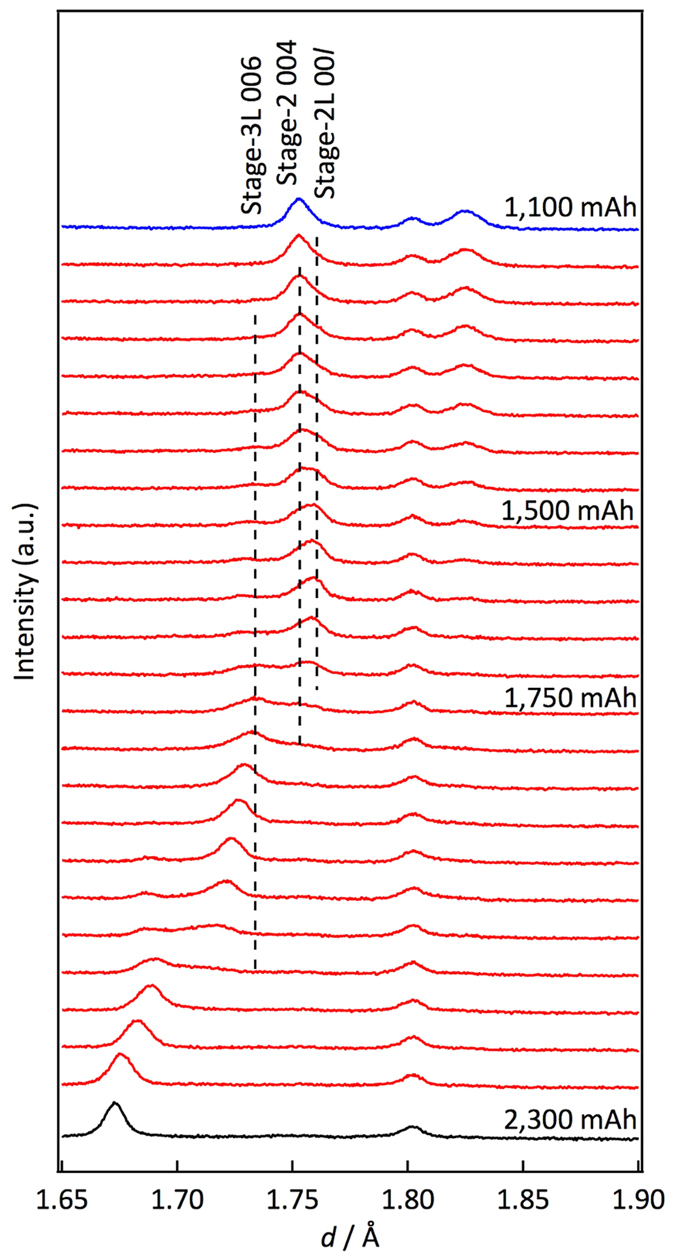
Two-phase reaction for stage 2 region during discharge process. One-dimensional profile of the diffraction patterns for carbon during the discharge process at the 0.05 C rate. The diffraction peaks of the 004 and 006 reflections are indicated in the figure. The peak splitting of the 004 reflection indicates that the phase changes proceed by two-phase coexistence regions for stage 2L and stage 2.

A kinetic difference between lithium insertion and extraction might cause changes in the rate characteristics for the charge and discharge processes. Figure 5 shows two-dimensional neutron diffraction profiles for the carbon 00*l* peak during the charge and discharge processes at the 0.05 C rate. In the charge process shown in Fig. 5(a,c), C (graphite) is transformed to stage 4L (LiC_24_) through a two-phase coexistence region. The stage 4L (LiC_24_) phase changes to a stage 3L (LiC_18_) phase with a continuous peak shift of the stage 3L (LiC_18_). The stage 2 (LiC_12_) phase is transformed to a stage 1 (LiC_6_) phase through a two-phase region; these phase changes are consistent with previous reports^9^,^19^,^20^. For the discharge process shown in Fig. 5(b,d), similar phase changes are observed, except for the stage 2/3 (LiC_12_/LiC_18_) region, in which peak shifts for both phases are observed. Although a continuous shift of the 002 reflection to higher *d*-values is observed for the stage 2 phase in the capacity range from 1,333 to 1,667 mAh, the higher order 004 reflection shown in Fig. 6 clearly indicates a peak separation due to the coexistence of two phases in the same capacity range. This corresponds to the presence of both stage 2 and stage 2L phases^33^. These results indicate that 2L structures exist only in the discharge process, which might be caused by slow lithium diffusion kinetics during the charge process. In the charge process, the stage 3L structure is likely to be directly transformed to the stage 2 structure without formation of the 2L phase because lithium diffusion in the 3L phase is slow under practical cell operation conditions. These observations are consistent with those indicated by electrochemical measurements^9^.

#### Non-equilibrium battery reactions in the anode

**Figure 7:**
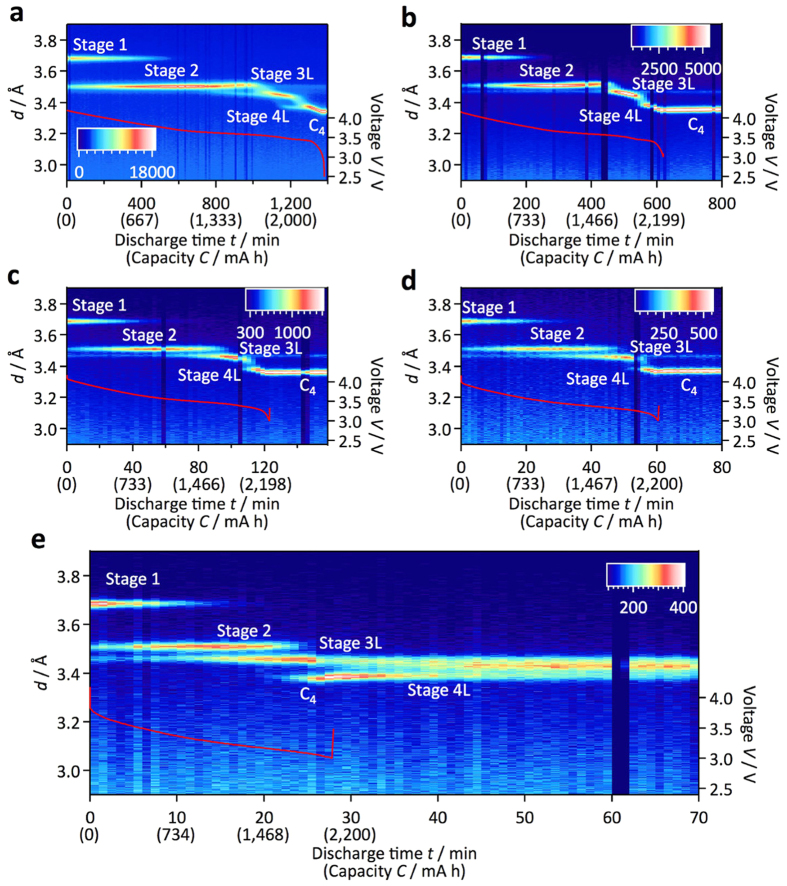
Phase changes during discharge experiments. Diffraction profiles of the carbon 00*l* peak during discharge experiments. The discharge rates were 0.05 (**a**), 0.1 (**b**), 0.5 (**c**), 1 (**d**), and 2 C (**e**). The voltage profiles of each discharge reaction are also indicated. The residual 3L phase exists during cycling above the 0.1 C rate without a change in peak position (see (**b**)), discharge rate: 0.1 C). The 4L (LiC_24_) phase gradually changes to the 3L (LiC_18_) phase after the end of the discharge (see (**e**)). The high current causes inhomogeneous lithium distribution in the electrode matrix.

During battery operation, reactions proceed inhomogeneously in the electrode matrix, particularly at high C rates. Figure 7 shows the C-rate dependence of the graphite diffraction profile with discharge. As the C rate increases from 0.05 to 0.1, the two-phase coexistence region of stages 1 (LiC_6_) and 2 (LiC_12_) increases in the initial discharge. A three-phase region of stages 1, 2, and 3L (LiC_18_) appears at the 1 C rate, and becomes dominant at the 2 C rate. These changes indicate an inhomogeneous reaction along the depth^11^ and in-plane^10^ directions in the electrode sheet and/or in the individual particles^9^,^34^, and clearly show that fast, homogeneous structural changes are difficult under high-current conditions.

Another important observation involves the residual 3L phase during cycling. Although the 3L phase exists in a small composition region at 0.05 C, this phase is continuously present above 0.1 C, without changes in its peak position. This indicates that once the 3L phase forms, it remains in the cells without participating in the reactions. The Daumas-Hérold domain model was proposed to explain the kinetics of lithium (de-)intercalation for graphite anodes^35^. Deintercalation of lithium is initiated through a stage 1 structure to form higher stage compounds consisting of a few tens of domains with staggered boundaries. Further deintercalation eventually results in the formation of residual lithium islands surrounded by large areas of deintercalated graphite interlayers, which kinetically prevent lithium (de-)intercalation of the host structure due to collapse of the interlayer space, and the lithium mobility sharply decreases^36^. Although the 3L phase observed at the end of discharge under high-rate operation in our experimental results is consistent with this phase change model, further work using a model electrode system is required to clarify the detailed mechanism. For discharge at the 2 C rate, lower cell voltages are observed than at other C rates. The stage 2 phase, with its higher electrode potential, is observed at an earlier stage of the discharge, which also indicates an inhomogeneous cell reaction.

In the previous section, the 2L structure was found to exist only during the discharge due to the slow lithium diffusion kinetics during the charge. This is also confirmed by the changing C rate in the discharge. The rate dependence of the phase change shown in Fig. 7 is replotted in Supplementary Fig. S3 to clearly show the phase change near the 2L/3L phase region. The 2L phase is observed at the 0.1 C rate, whereas this phase is not observed over a discharge rate of 0.5 C. The existence of the stage 2L phase is observed only for the low C-rate discharge, and the state 2 structure is directly transformed to the stage 3L structure at high current rates.

In addition to the rate dependence of the structural changes, it is important to note that the phase changed after the discharge process. The 4L (LiC_24_) phase gradually changes to the 3L (LiC_18_) phase after the end of the discharge. The relaxation process after the high current discharge indicates slow lithium diffusion kinetics in carbon or in the electrode matrix. Lithium diffusion in the 3L phase might be slower than that in the 4L phase, which would account for the slow kinetics and the relaxation process after the current has passed through the cell^37^. The high current discharge results in inhomogeneous lithium distribution in the electrode matrix, and provides the relaxation process after the cell operation.

#### Non-equilibrium battery reactions in the cathode

**Figure 8:**
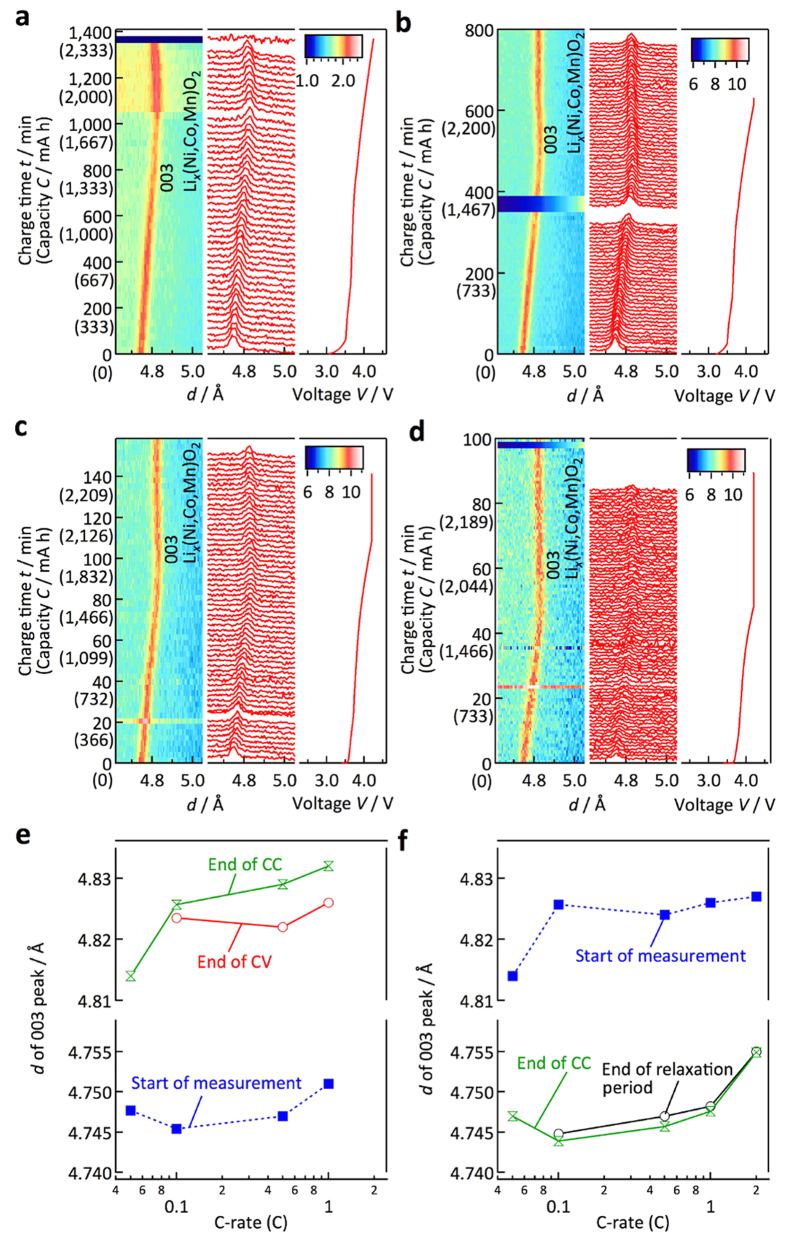
Structural changes of cathode material during practical battery operation. Graphical and one-dimensional profiles of the diffraction patterns for the cathode during charge reactions in (**a**–**d**). The corresponding profiles during discharge reactions are shown in Supplementary Fig. S4. The 003 diffraction peaks are indicated in the figure. The charge rates were 0.05 (**a**), 0.1 (**b**), 0.5 (**c**), and 1 C (**d**). The voltage profiles of the charge reactions are indicated for each diffraction profile. (**e**) *d*-values for the 003 reflection of the NMC cathode material in the initial state, after the end of the CC mode charge, and after the end of the CV mode charge. (**f**) *d*-values for the 003 reflection of the NMC cathode materials in the initial state, after the end of the CC discharge mode, and after the end of the relaxation time. The *d*_003_-value changes for all the data are indicated in Supplementary Fig. S5. These values are plotted both for the charge and discharge processes as a function of the C rate of the CC mode.

Figure 8 shows the graphical and one-dimensional diffraction profiles of the cathode for the charge process. With a charge by constant-current (CC) mode, the 003 peak shifts first to higher *d*-values, and then to lower, which is consistent with the reported *c*-axis changes with lithium deintercalation^23^. At 4.2 V, the CC mode charge was switched to a constant-voltage (CV) mode, in which the 003 peak still shifts with the charge. The *d*_003_-value changes are shown in Supplementary Fig. S5.

Figure 8(e,f) summarize the C-rate dependence of the *d*_003_-value changes. For the charge, the *d*-values are plotted for the initial state (before charge), after the CC charge, and after the CV charge. After the charge in CC mode, the *d*-values increase from around 4.745 to 4.81–4.83 Å, and larger *d*_003_-values are observed for the higher C-rate charges. This indicates that the cathode after the high current charge has a higher lithium content, which might be caused by large overpotentials for the high-current charge condition. The large overpotential for the high-current charge condition could mainly come from the negative electrode in the high-power 18650-type cell^38^. The CV charge after the CC mode might increase the lithium content. However, the *d*_003_-values at the end of the CV charge are still larger than those observed for the 0.05 C-rate charge, although the *d*_003_-values still decrease during the CV charge. The amount of lithium in the cathode after the 0.1–1 C-rate CC and CV charge is much higher than that observed at the 0.05 C rate. This indicates that the charge of our CV conditions was not sufficient to attain equilibrium (CV-mode conditions: the charge current was stopped after the current was less than 110 mA for the 0.5 and 1 C rates, and 70 mA for the 0.1 C rate).

For the discharge, the *d*_003_-values are plotted for the initial state (after charge), after the CC mode, and after a certain relaxation period. No changes are observed between the *d*-values after the CC discharge and after the relaxation period, indicating a homogeneous reaction in the cathode matrix. On the other hand, the *d*-value after the 2 C-rate discharge is larger than those of other C rates, indicating a smaller amount of lithium intercalated by the reaction. More importantly, the larger *d*_003_-values observed at both the initial and the final states for the high C rates suggest that the lithium composition ranges used for the reactions in the cathode and anode shift with the rate conditions. Previously, a narrowing of the composition range for the discharge process was detected with an increase of the reaction rate using *ex situ* XRD measurements^39^. However, to the best of our knowledge, there are no observations regarding the composition shifts for the battery reaction when the current density is increased. This is the first experimental evidence that the composition shifts for 18650 full-cell experiments. The operando observations of the 18650 cell detected that the reaction at a high current drain was different from those at low current drains.

## Discussion

We developed an *in situ* and operando analysis of battery reactions using the SPICA TOF neutron diffractometer, and our method was found to be suitable for the detection of non-homogeneous and non-equilibrium reactions under practical battery operation conditions.

The operando observations of the 18650 cell at a high current rate revealed inhomogeneous reactions in the electrode matrix and a relaxation process that occurs after the high current drain discharge. The results obtained are summarized as follows. Residual electrode components that did not participate in the battery reaction were found to exist under high C-rate charge and discharge conditions. A relaxation process was observed after the high C-rate current passed through the cell, which was caused by the inhomogeneous reactions in the electrode matrix. In addition to the inhomogeneous reactions taking place at high current rates, the mechanism changes and a kinetic difference is observed between the charge and discharge processes. The method also allowed the determination of the lithium concentration directly in the cell. The composition ranges used for the charge and discharge reactions depend on the C-rate conditions. The neutron intensity used in our experimental system was sufficient to determine the lithium composition in the electrodes during battery operation, which could shed light on lithium concentration changes during cycling.

The advantages of the TOF diffractometer are as follows^18^,^40^,^41^. (i) It allows the simultaneous collection of diffraction data over a wide scattering vector (Q) range, which is suitable for time-resolved analysis. Accumulating diffraction data with high-intensity neutrons makes accurate structural analysis possible under non-equilibrium conditions in the electrode matrix at each charge or discharge reaction step. (ii) Neutrons penetrate the stainless-steel cans used for commercial cells, which enables the collection of diffraction data with sufficient accuracy to determine the structures of the electrode materials under high C-rate conditions. (iii) Neutrons can detect light elements and enable the determination of the position and concentration of lithium in the cell. (iv) Large-scale cells contain large amounts of electrode materials, which provide diffraction patterns of high quality and high intensity. This suppresses the disadvantages of inelastic scattering and the effects of absorption by proton-containing organic electrolytes.

Our neutron observations using the TOF diffractometer provided valuable information about battery reactions under practical conditions. This type of information is important for the total understanding of battery processes in practical cells. Although the precise reaction mechanisms for each battery component and at the electrode/electrolyte interface can be determined by many other *in situ* experimental techniques based on neutron and X-ray scattering, our experimental tool should be the first step in analysing a battery under practical usage conditions. The *in situ*, operando observations using TOF neutron diffraction provide one of the best methods for detecting reaction mechanisms under practical battery operation conditions.

## Methods

### SPICA diffractometer and experimental setup for battery operation

The high neutron intensity for the operando measurements was achieved with the SPICA diffractometer installed at BL09 in the 1^st^ experimental hall of the Materials and Life Science Experimental Facility (MLF) of the Japan Proton Accelerator Research Complex (J-PARC). The sample position was 52 m from the poisoned and decoupled moderator. The use of the poisoned moderator selected for the SPICA allowed the generation of symmetrical peak profiles for the diffractions, which enabled the extraction of the strain information from the electrode materials. In addition, a focusing supermirror used for the neutron guide tube increased the number of neutrons that reached the sample position, and thus increased the diffraction intensities. An elliptical guide with off-focusing in the vertical and horizontal planes was one of the candidates for focusing. After several elliptical guide designs were simulated using McStas^42^, the final design of the supermirror guide, which has a mirror coating that gradually changes from *m* = 3 to 6 at the posterior half of the guide, was adopted^18^. This experimental setup allowed a battery size of up to 1.0 m in diameter for the *in situ* measurements, with an incident neutron beam size of 20 (width) × 40 (height) mm. We used 18650-type cells in the present study. The upper 10 mm of the cylindrical cell was used for fixing the cell and connecting to the cables of the electrochemical instrument. Therefore, a region that was 55 mm in height from the bottom of the cell was irradiated by the neutron beam in these experiments.

### Experimental process for *in situ* and operando observations of the commercial 18650 cell

For the *in situ* measurements, a high-power 18650-type Li-ion rechargeable battery (cathode: Li(Ni,Mn,Co)O_2_, anode: graphite, normal voltage: 3.6 V, standard capacity 2,300 mAh) was used. *In situ* neutron diffraction data were collected at cycle rates of 0.05, 0.1, 0.5, 1, and 2 C during discharging and 0.05, 0.1, 0.5, and 1 C during charging. The electrochemical measurements during the *in situ* measurements were performed with a VSP-300 potentiostat/galvanostat with four 10 A booster board kits (Bio-Logic Science Instruments). The high-power cell with a nominal capacity of 2,300 mAh was cycled at 0.1, 0.22, 1.1, and 2.2 A (theoretically equivalent to 0.05, 0.1, 0.5, and 1 C, respectively) in the CC/CV-charging and CC-discharging modes. A 4.4 A current (theoretically equivalent to 2 C) during CC discharging was applied to investigate faster electrode reactions.

*In situ* neutron diffraction patterns for the 18650-type cells were also obtained at room temperature on the special-environment SPICA^18^ powder diffractometer. All diffraction patterns were collected using detectors in the range 10 < 2*θ* < 175°. Data from the 90° bank (70 < 2*θ* < 110°) were mainly used to characterize the crystal structures of the electrode materials. The SPICA continually and individually acquired all the diffracted neutrons from the cells (with their positions and times-of-flight) which reached the detectors during the charge and discharge processes. Therefore, the time divisions in a diffraction pattern could be changed, even after measurement, by the data reduction techniques. “Time-divided” diffraction data were sliced by the time of appropriate lithium ion transfer. The structural parameters of the cathode and anode materials were characterized and refined with the Z-Rietveld program^43^,^44^. The plastic cover films on the cells were removed before the experiments to reduce the background. The background levels were not particularly high, even though materials such as hydrogen atoms that have high incoherent scattering lengths were present. However, the current collector metals and battery housing were fitted using individual profile parameters because of their highly preferred orientations.

### Automatic analysis of big data for the diffraction patterns

The diffraction data obtained from the time-division diffraction analysis provides sequential diffraction profiles of the electrode materials. Although this reveals information about the phase changes during the electrochemical process, more precise information based on the lattice parameters, phase ratios, and ion distributions in the electrodes is obtained by the Rietveld analysis. However, sequential data collection provides a large number of data sets, which is difficult for a manual refinement process. The Z-Rietveld analysis developed in the present study provides an automatic diffraction analysis routine suitable for battery materials and *in situ*, operando analysis, which is composed of multiple data sets based on time-resolved data collection, data sets for pole figure data analysis, and temperature-dependence data collection procedures. Figure 1c shows a flowchart of the data analysis routine and refined diffraction patterns. The data analysis procedure includes two steps: data reduction for the structural analysis and refinement of the crystal structures of both the cathode and anode electrodes. The data accumulated during charge and discharge at the 1/20 C rate, for example, provides a data set with a size of 120 Gbyte, which corresponds to approximately 30 G diffracted neutrons recorded by the SPICA data acquisition system. The huge size of this diffraction data set was reduced by the sequential data accumulation routine, and converted into diffraction data for short time periods to analyse each charge and discharge process.

## Additional Information

**How to cite this article**: Taminato, S. *et al*. Real-time observations of lithium battery reactions—operando neutron diffraction analysis during practical operation. *Sci. Rep.* **6**, 28843; doi: 10.1038/srep28843 (2016).

## Supplementary Material

Supplementary Information
