# Supplementary material for: Real-time observations of lithium battery reactions—operando neutron diffraction analysis during practical operation
Source: Sci Rep. 2016 Jun 30;6:28843. doi: 10.1038/srep28843 (PMC4928065; doi:10.1038/srep28843)
Supplement: Supplementary Information [file srep28843-s1.pdf]

Supplementary Information

Real-time observations of lithium battery reactions—operando neutron diffraction analysis during practical operation

Sou Taminato[1], Masao Yonemura[2,3], Shinya Shiotani[4], Takashi Kamiyama[2,3], Shuki Torii[2], Miki Nagao[2], Yoshihisa Ishikawa[2], Kazuhiro Mori[5], Toshiharu Fukunaga[5], Yohei Onodera[5], Takahiro Naka[4], Makoto Morishima[4], Yoshio Ukyo[4], Dyah Sulistyanintyas Adipranoto[4], Hajime Arai[4], Yoshiharu Uchimoto[6], Zempachi Ogumi[4], Kota Suzuki[1], Masaaki Hirayama[1] and Ryoji Kanno[1]*

[1] Department of Chemical Science and Engineering, School of Materials and Chemical Technology, Tokyo Institute of Technology, 4259 Nagatsuta, Midori-ku, Yokohama 226-8502, Japan

[2] Institute of Materials Structure Science, High Energy Accelerator Research Organization, 1-1 Oho, Tsukuba, Ibaraki 305-0801, Japan

[3] Sokendai (The Graduate University for Advanced Studies), Shirakata 203-1, Tokai, Naka 319-1106, Japan

[4] Office of Society-Academia Collaboration for Innovation, Kyoto University, Uji-shi, Kyoto 611-0011, Japan

[5] Research Reactor Institute, Kyoto University, Kumatori-cho, Sennan-gun, Osaka 590-0494, Japan

[6] Graduate School of Human and Environmental Studies, Kyoto University, Sakyo-ku, Kyoto 606-8501, Japan

* Corresponding author: Department of Electronic Chemistry, Interdisciplinary Graduate School of Science and Engineering, Tokyo Institute of Technology, 4259 Nagatsuta-cho, Midori-ku, Yokohama 226-8502, Japan. Tel.: +81 45 924 5401; fax: +81 45 924 5401

E-mail address: kanno@echem.titech.ac.jp (R. Kanno).

Supplementary methods

Detection of lithium concentration in the cell

For the determination of lithium concentration, a custom designed 18650-type Li-ion rechargeable battery was used. The cell consists of $Li_x(Ni_{1/3}Co_{1/3}Mn_{1/3})O_2$ cathode and graphite anode materials with 1 mol dm^{-3} $LiPF_6$ in a 3:7 mixture of ethylene carbonate and dimethyl carbonate as the electrolyte. Aluminium and copper foils are used as a current collector. The customized cell with a nominal capacity of 1,800 mAh was cycled at 0.18 A (theoretically equivalent to 0.1 C) in the CC/CV-charging and CC-discharging modes.

Supplementary Table S1 summarises the refinement results for the neutron diffraction experiments conducted at the 0.1 C rate. For the refinement of the cathode material, the thermal parameters B of the all sites were fixed, corresponding to the common values for the lithium metal oxides, $B_{\mathrm{Li}} = 0.9$, $B_{\mathrm{Ni,Co,Mn}} = 0.3$, $B_{\mathrm{O}} = 0.5$, and $B_{\mathrm{C}} = 0.3$[1,2]. Before the operando refinement of the NMC cathode, the Li/Ni mixing in the NMC cathode was refined using another cell with the same cell configuration. This refinement demonstrated no Li/Ni mixing in the NMC layered cathode. The site occupation parameters g were fixed at 0.333 and 1.0 for the transition metal and oxygen atoms, respectively. The lattice parameters of the a and c axes, the site occupation parameters of the lithium atoms on the 3b sites, and the fractional coordinate z of the oxygen atoms on the 6c sites (0, 0, z) were refined. For the refinement of the anode materials, only the lattice parameters and mass ratios of the two carbon materials were refined. The structural parameters of copper and aluminium as current collectors and α-type iron from the casing used the following respective models. For aluminium and copper: space group $Fm\bar{3}m$, with Al and Cu at 4a (0, 0, 0) sites[3,4]. For α-type iron: space group $Im\bar{3}m$, with Fe at 2a (0, 0, 0) sites[5]. The lattice parameters were only refined using the above models in the initial state of the cell, and were fixed in the subsequent refinements. The thermal parameters and site occupancies of these metal compounds were fixed as 0.5 and 1.0, respectively.

Supplementary Table S1. Rietveld refinement results for neutron diffraction patterns of 18650-type Li-ion cells in discharged states of (a) 0–61.7, (b) 61.7–123.3, (c) 123.3–185, (d) 185–246.7, (e) 246.7–308.3, (f) 308.3–370, (g) 370–431.7, (h) 555–616.7, (i) 616.7–678.3, (j) 678.3–740, (k) 740–801.7, and (l) 801.7–863.3 mAh.

a

Atom	Site	*g*	*x*	*y*	*z*	B/Å^2
$Li_xNi_{1/3}Co_{1/3}Mn_{1/3}O_2$						
Li(1)	3*b*	0.344(8)	0	0	1/2	0.9
Ni(1)	3*a*	0.333	0	0	0	0.3
Co(1)	3*a*	0.333	0	0	0	0.3
Mn(1)	3*a*	0.333	0	0	0	0.3
O(1)	6*c*	1	0	0	0.25912(9)	0.5
LiC_6						
Li(1)	1*a*	1	0	0	0	0.9
C(1)	6*k*	1	0	0.333	1/2	0.3
LiC_{12}						
Li(1)	1*a*	1	0	0	0	0.9
C(1)	12*n*	1	0	0.333	0.25	0.3

For $Li_xNi_{1/3}Co_{1/3}Mn_{1/3}O_2$: Space group $R\bar{3}m$ (166), a = 2.81371(8) Å, c = 14.4890(10) Å, Phase fraction = 17.59%, R_B = 10.71%, R_F = 14.95%. For LiC_6: Space group *P6/mmm* (191), a = 4.31178(7) Å, c = 3.69571(18) Å, Phase fraction = 10.20%, R_B = 2.73%, R_F = 8.68%. For LiC_{12}: Space group *P6/mmm* (191), a = 4.29050(7) Å, c = 7.02506(8) Å, Phase fraction = 3.87%, R_B = 5.95%, R_F = 10.59%. R_{wp} = 1.70%, R_p = 1.26%, R_e = 0.36%, S (= R_{wp}/R_e) = 4.75.

B

Atom	Site	*g*	*x*	*y*	*z*	*B*/Å^2
$Li_xNi_{1/3}Co_{1/3}Mn_{1/3}O_2$						
Li(1)	3*b*	0.429(8)	0	0	1/2	0.9
Ni(1)	3*a*	0.333	0	0	0	0.3
Co(1)	3*a*	0.333	0	0	0	0.3
Mn(1)	3*a*	0.333	0	0	0	0.3
O(1)	6*c*	1	0	0	0.26073(7)	0.5
LiC_6						
Li(1)	1*a*	1	0	0	0	0.9
C(1)	6*k*	1	0	0.333	1/2	0.3
LiC_{12}						
Li(1)	1*a*	1	0	0	0	0.9
C(1)	12*n*	1	0	0.333	0.25	0.3

For $Li_xNi_{1/3}Co_{1/3}Mn_{1/3}O_2$: Space group $R\bar{3}m$ (166), a = 2.81370(6) Å, c = 14.5047(10) Å, Phase fraction = 17.59%, R_B = 16.74%, R_F = 13.60%. For LiC_6: Space group *P6/mmm* (191), a = 4.31257(3) Å, c = 3.69373(7) Å, Phase fraction = 9.73%, R_B = 4.36%, R_F = 9.98%. For LiC_{12}: Space group *P6/mmm* (191), a = 4.28919(3) Å, c = 7.0376(3) Å, Phase fraction = 4.34%, R_B = 10.25%, R_F = 11.23%. R_{wp} = 1.78%, R_p = 1.36%, R_e = 0.36%, S = 4.95.

c

Atom	Site	*g*	*x*	*y*	*z*	*B*/Å^2
$Li_xNi_{1/3}Co_{1/3}Mn_{1/3}O_2$						
Li(1)	3*b*	0.373(9)	0	0	1/2	0.9
Ni(1)	3*a*	0.333	0	0	0	0.3
Co(1)	3*a*	0.333	0	0	0	0.3
Mn(1)	3*a*	0.333	0	0	0	0.3
O(1)	6*c*	1	0	0	0.26087(8)	0.5
LiC_6						
Li(1)	1*a*	1	0	0	0	0.9
C(1)	6*k*	1	0	0.333	1/2	0.3
LiC_{12}						
Li(1)	1*a*	1	0	0	0	0.9
C(1)	12*n*	1	0	0.333	0.25	0.3

For $Li_xNi_{1/3}Co_{1/3}Mn_{1/3}O_2$: Space group $R\bar{3}m$ (166), a = 2.81401(6) Å, c = 14.5062(11) Å, Phase fraction = 17.59%, R_B = 9.10%, R_F = 14.65%. For LiC_6: Space group *P6/mmm* (191), a = 4.31173(7) Å, c = 3.69544(18) Å, Phase fraction = 9.05%, R_B = 3.74%, R_F = 7.04%. For LiC_{12}: Space group *P6/mmm* (191), a = 4.28813(6) Å, c = 7.0487(6) Å, Phase fraction = 5.02%, R_B = 6.51%, R_F = 10.63%. R_{wp} = 1.61%, R_p = 1.21%, R_e = 0.38%, S = 4.19.

d

Atom	Site	*g*	*x*	*y*	*z*	*B*/Å^2
$Li_xNi_{1/3}Co_{1/3}Mn_{1/3}O_2$						
Li(1)	3*b*	0.504(10)	0	0	1/2	0.9
Ni(1)	3*a*	0.333	0	0	0	0.3
Co(1)	3*a*	0.333	0	0	0	0.3
Mn(1)	3*a*	0.333	0	0	0	0.3
O(1)	6*c*	1	0	0	0.26013(7)	0.5
LiC_6						
Li(1)	1*a*	1	0	0	0	0.9
C(1)	6*k*	1	0	0.333	1/2	0.3
LiC_{12}						
Li(1)	1*a*	1	0	0	0	0.9
C(1)	12*n*	1	0	0.333	0.25	0.3

For $Li_xNi_{1/3}Co_{1/3}Mn_{1/3}O_2$: Space group $R\bar{3}m$ (166), a = 2.81454(6) Å, c = 14.5046(10) Å, Phase fraction = 17.59%, R_B = 9.69%, R_F = 15.62%. For LiC_6: Space group *P6/mmm* (191), a = 4.31143(8) Å, c = 3.6955(2) Å, Phase fraction = 8.33%, R_B = 5.27%, R_F = 8.32%. For LiC_{12}: Space group *P6/mmm* (191), a = 4.28829(5) Å, c = 7.0378(5) Å, Phase fraction = 5.74%, R_B = 6.63%, R_F = 11.42%. R_{wp} = 1.65%, R_p = 1.23%, R_e = 0.36%, S = 4.54.

e

Atom	Site	*g*	*x*	*y*	*z*	*B*/Å^2
$Li_xNi_{1/3}Co_{1/3}Mn_{1/3}O_2$						
Li(1)	3*b*	0.432(8)	0	0	1/2	0.9
Ni(1)	3*a*	0.333	0	0	0	0.3
Co(1)	3*a*	0.333	0	0	0	0.3
Mn(1)	3*a*	0.333	0	0	0	0.3
O(1)	6*c*	1	0	0	0.25966(7)	0.5
LiC_6						
Li(1)	1*a*	1	0	0	0	0.9
C(1)	6*k*	1	0	0.333	1/2	0.3
LiC_{12}						
Li(1)	1*a*	1	0	0	0	0.9
C(1)	12*n*	1	0	0.333	0.25	0.3

For $Li_xNi_{1/3}Co_{1/3}Mn_{1/3}O_2$: Space group $R\bar{3}m$ (166), a = 2.81520(6) Å, c = 14.5048(10) Å, Phase fraction = 17.59%, R_B = 11.78%, R_F = 15.75%. For LiC_6: Space group *P6/mmm* (191), a = 4.31108(9) Å, c = 3.6964(2) Å, Phase fraction = 7.57%, R_B = 6.11%, R_F = 9.61%. For LiC_{12}: Space group *P6/mmm* (191), a = 4.28801(5) Å, c = 7.0384(5) Å, Phase fraction = 6.50%, R_B = 6.42%, R_F = 11.65%. R_{wp} = 1.67%, R_p = 1.26%, R_e = 0.37%, S = 4.50.

f

Atom	Site	g	x	y	z	$B/Å^2$
$Li_xNi_{1/3}Co_{1/3}Mn_{1/3}O_2$						
Li(1)	3b	0.481(8)	0	0	1/2	0.9
Ni(1)	3a	0.333	0	0	0	0.3
Co(1)	3a	0.333	0	0	0	0.3
Mn(1)	3a	0.333	0	0	0	0.3
O(1)	6c	1	0	0	0.25894(7)	0.5
LiC_6						
Li(1)	1a	1	0	0	0	0.9
C(1)	6k	1	0	0.333	1/2	0.3
LiC_{12}						
Li(1)	1a	1	0	0	0	0.9
C(1)	12n	1	0	0.333	0.25	0.3

For $Li_xNi_{1/3}Co_{1/3}Mn_{1/3}O_2$: Space group $R\bar{3}m$ (166), a = 2.81481(6) Å, c = 14.5136(10) Å, Phase fraction = 17.59%, R_B = 11.62%, R_F = 16.09%. For LiC_6: Space group: *P6/mmm* (191), a = 4.31053(11) Å, c = 3.6979(2) Å, Phase fraction = 6.78%, R_B = 6.47%, R_F = 9.12%. For LiC_{12}: Space group *P6/mmm* (191), a = 4.28874(4) Å, c = 7.0268(3) Å, Phase fraction = 7.29%, R_B = 6.55%, R_F = 11.24%. R_{wp} = 1.67%, R_p = 1.26%, R_e = 0.36%, S = 4.64.

g

Atom	Site	g	x	y	z	B/Å^2
$Li_xNi_{1/3}Co_{1/3}Mn_{1/3}O_2$						
Li(1)	3*b*	0.503(8)	0	0	1/2	0.9
Ni(1)	3*a*	0.333	0	0	0	0.3
Co(1)	3*a*	0.333	0	0	0	0.3
Mn(1)	3*a*	0.333	0	0	0	0.3
O(1)	6*c*	1	0	0	0.25954(7)	0.5
LiC_6						
Li(1)	1*a*	1	0	0	0	0.9
C(1)	6*k*	1	0	0.333	1/2	0.3
LiC_{12}						
Li(1)	1*a*	1	0	0	0	0.9
C(1)	12*n*	1	0	0.333	0.25	0.3

For $Li_xNi_{1/3}Co_{1/3}Mn_{1/3}O_2$: Space group $R\bar{3}m$ (166), a = 2.81597(6) Å, c = 14.5053(10) Å, Phase fraction = 17.59%, R_B = 9.99%, R_F = 15.41%. For LiC_6: Space group *P6/mmm* (191), a = 4.31029(13) Å, c = 3.6988(3) Å, Phase fraction = 5.99%, R_B = 6.53%, R_F = 8.75%. For LiC_{12}: Space group *P6/mmm* (191), a = 4.28796(4) Å, c = 7.0325(4) Å, Phase fraction = 8.08%, R_B = 5.88%, R_F = 10.49%. R_{wp} = 1.67%, R_p = 1.25%, R_e = 0.36%, S = 4.60.

h

Atom	Site	*g*	*x*	*y*	*z*	*B*/Å^2
$Li_xNi_{1/3}Co_{1/3}Mn_{1/3}O_2$						
Li(1)	3*b*	0.577(8)	0	0	1/2	0.9
Ni(1)	3*a*	0.333	0	0	0	0.3
Co(1)	3*a*	0.333	0	0	0	0.3
Mn(1)	3*a*	0.333	0	0	0	0.3
O(1)	6*c*	1	0	0	0.25905(7)	0.5
LiC_6						
Li(1)	1*a*	1	0	0	0	0.9
C(1)	6*k*	1	0	0.333	1/2	0.3
LiC_{12}						
Li(1)	1*a*	1	0	0	0	0.9
C(1)	12*n*	1	0	0.333	0.25	0.3

For $Li_xNi_{1/3}Co_{1/3}Mn_{1/3}O_2$: Space group $R\bar{3}m$ (166), a = 2.81900(6) Å, c = 14.4838(10) Å, Phase fraction = 17.59%, R_B = 11.57%, R_F = 15.41%. For LiC_6: Space group *P6/mmm* (191), a = 4.3068(2) Å, c = 3.7061(6) Å, Phase fraction = 3.55%, R_B = 10.41%, R_F = 13.08%. For LiC_{12}: Space group *P6/mmm* (191), a = 4.28693(3) Å, c = 7.0309(3) Å, Phase fraction = 10.52%, R_B = 4.43%, R_F = 13.17%. R_{wp} = 1.80%, R_p = 1.29%, R_e = 0.36%, S = 4.98.

i

Atom	Site	*g*	*x*	*y*	*z*	B/Å^2
$Li_xNi_{1/3}Co_{1/3}Mn_{1/3}O_2$						
Li(1)	3*b*	0.549(8)	0	0	1/2	0.9
Ni(1)	3*a*	0.333	0	0	0	0.3
Co(1)	3*a*	0.333	0	0	0	0.3
Mn(1)	3*a*	0.333	0	0	0	0.3
O(1)	6*c*	1	0	0	0.25842(7)	0.5
LiC_6						
Li(1)	1*a*	1	0	0	0	0.9
C(1)	6*k*	1	0	0.333	1/2	0.3
LiC_{12}						
Li(1)	1*a*	1	0	0	0	0.9
C(1)	12*n*	1	0	0.333	0.25	0.3

For $Li_xNi_{1/3}Co_{1/3}Mn_{1/3}O_2$: Space group $R\bar{3}m$ (166), a = 2.82023(5) Å, c = 14.4760(9) Å, Phase fraction = 17.59%, R_B = 15.73%, R_F = 16.61%. For LiC_6: Space group *P6/mmm* (191), a = 4.3070(2) Å, c = 3.7062(6) Å, Phase fraction = 2.77%, R_B = 13.98%, R_F = 13.88%. For LiC_{12}: Space group *P6/mmm* (191), a = 4.28630(2) Å, c = 7.0324(2) Å, Phase fraction = 11.30%, R_B = 3.75%, R_F = 11.19%. R_{wp} = 2.05%, R_p = 1.40%, R_e = 0.36%, S = 5.66.

j

Atom	Site	g	x	y	z	B/Å^2
$Li_xNi_{1/3}Co_{1/3}Mn_{1/3}O_2$						
Li(1)	3*b*	0.602(8)	0	0	1/2	0.9
Ni(1)	3*a*	0.333	0	0	0	0.3
Co(1)	3*a*	0.333	0	0	0	0.3
Mn(1)	3*a*	0.333	0	0	0	0.3
O(1)	6*c*	1	0	0	0.25828(7)	0.5
LiC_6						
Li(1)	1*a*	1	0	0	0	0.9
C(1)	6*k*	1	0	0.333	1/2	0.3
LiC_{12}						
Li(1)	1*a*	1	0	0	0	0.9
C(1)	12*n*	1	0	0.333	0.25	0.3

For $Li_xNi_{1/3}Co_{1/3}Mn_{1/3}O_2$: Space group $R\bar{3}m$ (166), a = 2.82210(5) Å, c = 14.4594(9) Å, Phase fraction = 17.59%, R_B = 13.06%, R_F = 15.02%. For LiC_6: Space group *P6/mmm* (191), a = 4.2980(5) Å, c = 3.7101(11) Å, Phase fraction = 2.19%, R_B = 15.79%, R_F = 15.12%. For LiC_{12}: Space group *P6/mmm* (191), a = 4.28589(3) Å, c = 7.0280(3) Å, Phase fraction = 11.88%, R_B = 4.14%, R_F = 13.29%. R_{wp} = 2.00%, R_p = 1.37%, R_e = 0.36%, S = 5.50.

k

Atom	Site	*g*	*x*	*y*	*z*	*B*/Å^2
$Li_xNi_{1/3}Co_{1/3}Mn_{1/3}O_2$						
Li(1)	3*b*	0.647(9)	0	0	1/2	0.9
Ni(1)	3*a*	0.333	0	0	0	0.3
Co(1)	3*a*	0.333	0	0	0	0.3
Mn(1)	3*a*	0.333	0	0	0	0.3
O(1)	6*c*	1	0	0	0.25849(7)	0.5
LiC_6						
Li(1)	1*a*	1	0	0	0	0.9
C(1)	6*k*	1	0	0.333	1/2	0.3
LiC_{12}						
Li(1)	1*a*	1	0	0	0	0.9
C(1)	12*n*	1	0	0.333	0.25	0.3

For $Li_xNi_{1/3}Co_{1/3}Mn_{1/3}O_2$: Space group $R\bar{3}m$ (166), a = 2.82306(6) Å, c = 14.4512(11) Å, Phase fraction = 17.59%, R_B = 11.43%, R_F = 17.62%. For LiC_6: Space group *P6/mmm* (191), a = 4.2799(4) Å, c = 3.7127(6) Å, Phase fraction = 1.44%, R_B = 11.92%, R_F = 20.06%. For LiC_{12}: Space group *P6/mmm* (191), a = 4.28612(5) Å, c = 7.0281(3) Å, Phase fraction = 12.63%, R_B = 4.88%, R_F = 14.34%. R_{wp} = 2.22%, R_p = 1.48%, R_e = 0.38%, S = 5.89.

l

Atom	Site	*g*	*x*	*y*	*z*	B/Å^2
$Li_xNi_{1/3}Co_{1/3}Mn_{1/3}O_2$						
Li(1)	3*b*	0.643(8)	0	0	1/2	0.9
Ni(1)	3*a*	0.333	0	0	0	0.3
Co(1)	3*a*	0.333	0	0	0	0.3
Mn(1)	3*a*	0.333	0	0	0	0.3
O(1)	6*c*	1	0	0	0.26072(8)	0.5
LiC_6						
Li(1)	1*a*	1	0	0	0	0.9
C(1)	6*k*	1	0	0.333	1/2	0.3
LiC_{12}						
Li(1)	1*a*	1	0	0	0	0.9
C(1)	12*n*	1	0	0.333	0.25	0.3

For $Li_xNi_{1/3}Co_{1/3}Mn_{1/3}O_2$: Space group $R\bar{3}m$ (166), a = 2.82494(6) Å, c = 14.4344(8) Å, Phase fraction = 17.59%, R_B = 11.78%, R_F = 17.27%. For LiC_6: Space group *P6/mmm* (191), a = 4.2771(5) Å, c = 3.7124(8) Å, Phase fraction = 0.96%, R_B = 28.45%, R_F = 23.24%. For LiC_{12}: Space group *P6/mmm* (191), a = 4.28562(4) Å, c = 7.0266(3) Å, Phase fraction = 13.11%, R_B = 3.72%, R_F = 17.41%. R_{wp} = 2.26%, R_p = 1.46%, R_e = 0.36%, S = 6.23.

Supplementary Figures

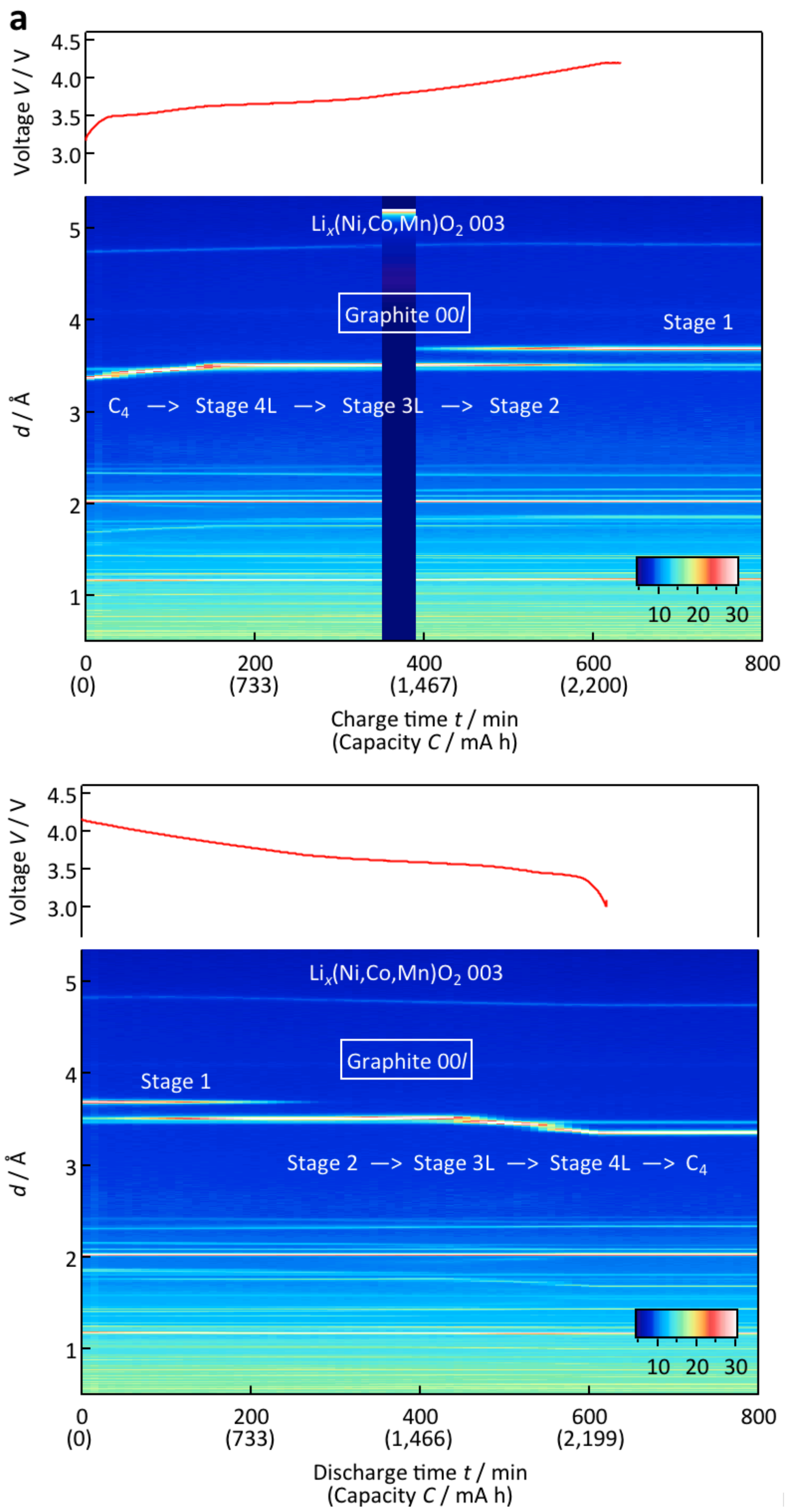

Supplementary Figure S1a

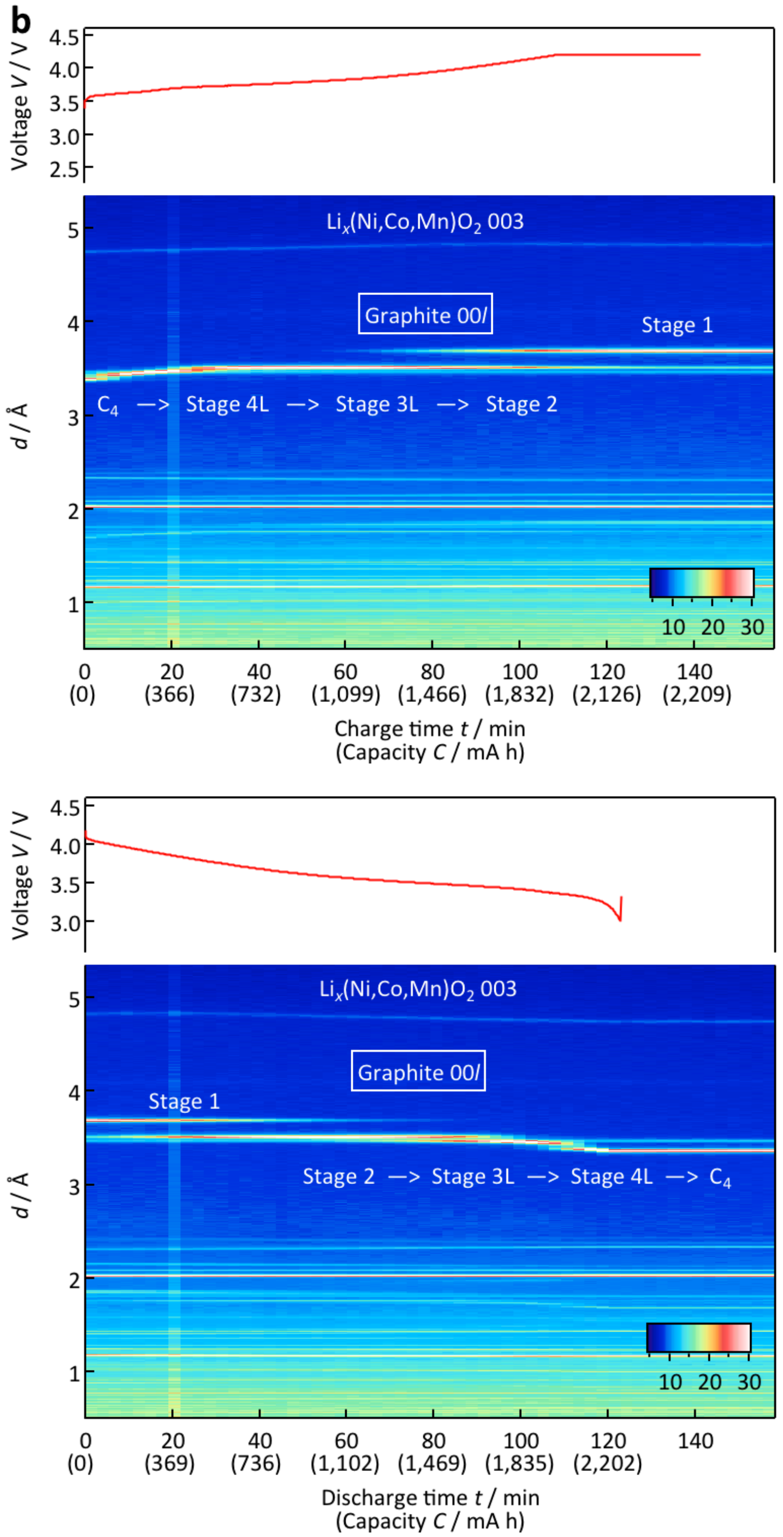

Supplementary Figure S1b

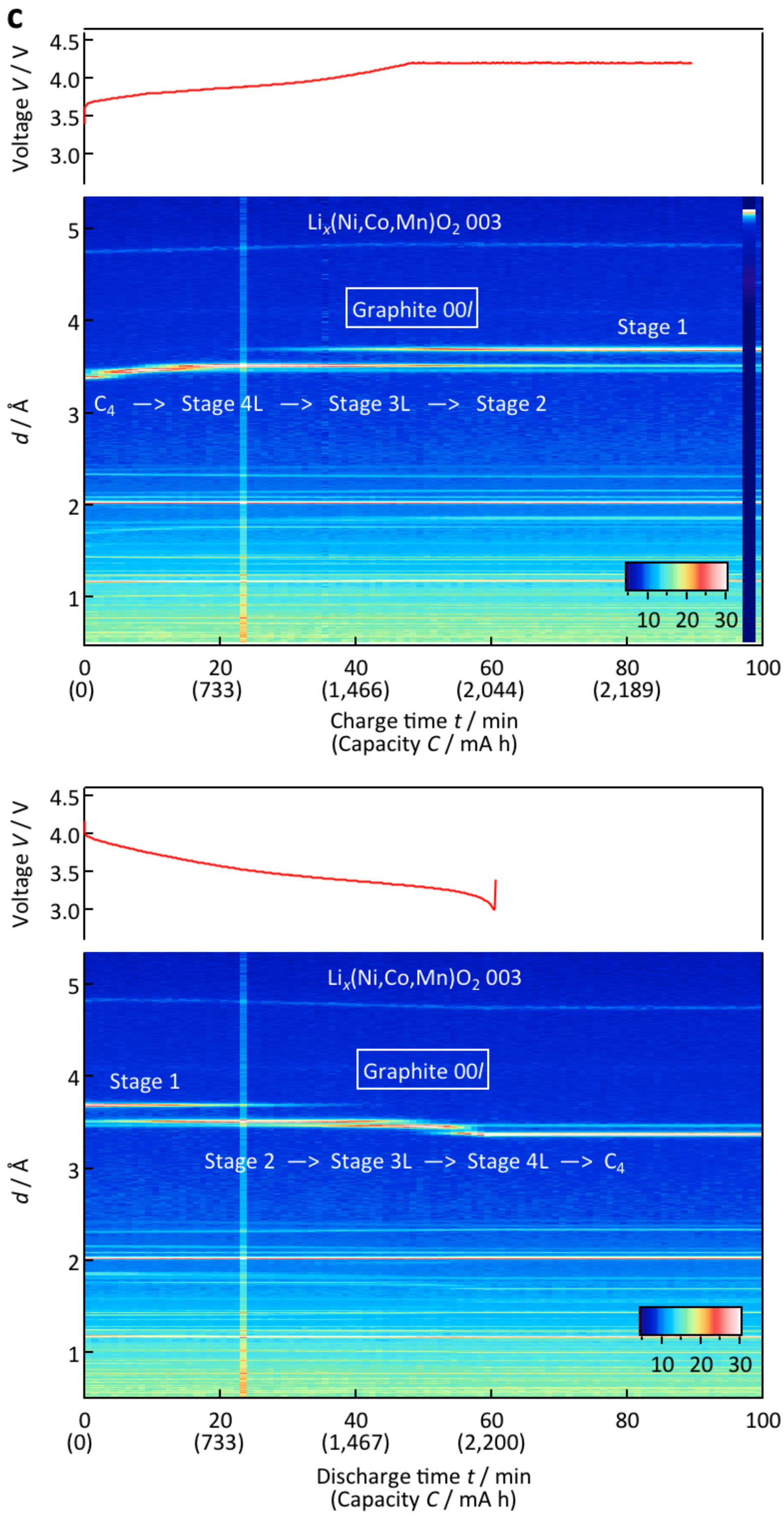

Supplementary Figure S1c

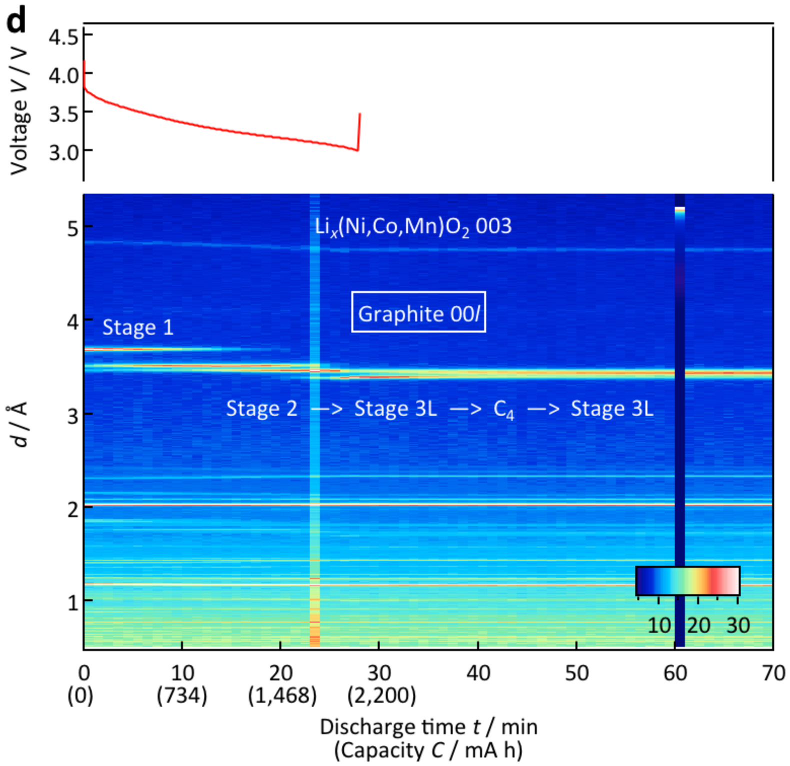

Supplementary Figure S1d

Supplementary Figure S1 | Neutron diffraction profiles during the battery reaction. Total diffraction profiles of the batteries during charge and discharge experiments. The charge and discharge rates were 0.1 (a), 0.5 (b), 1 (c), and 2 C (d). The profile changes at the 2 C rate are only indicated for the discharge process because the charge reaction was conducted at the 1 C rate. The voltage profiles of the charge and discharge reactions are indicated for each diffraction profile.

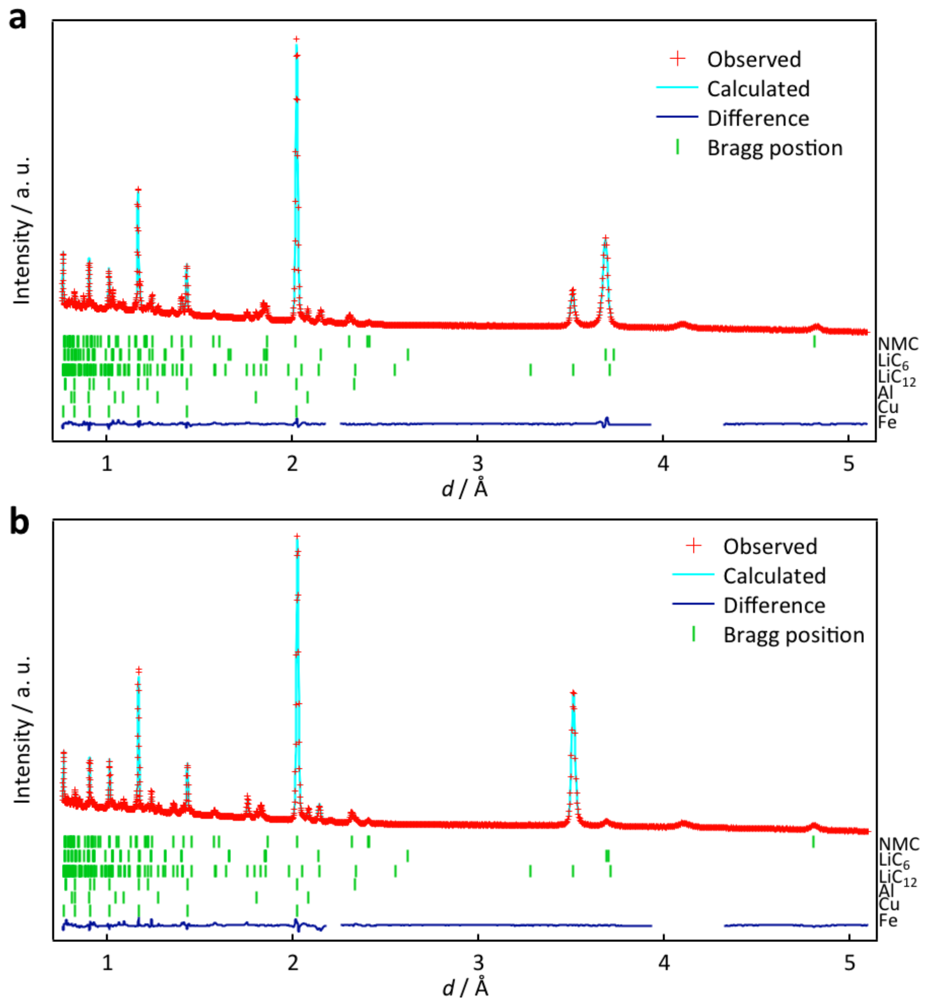

Supplementary Figure S2 | Rietveld-refined neutron diffraction patterns. Rietveld refinement patterns for the NMC cathode during the charge and discharge experiments. The refinement patterns are shown for the initial (a) and post-discharge (b) states. Green dashes indicate the positions of Bragg reflections for the battery components.

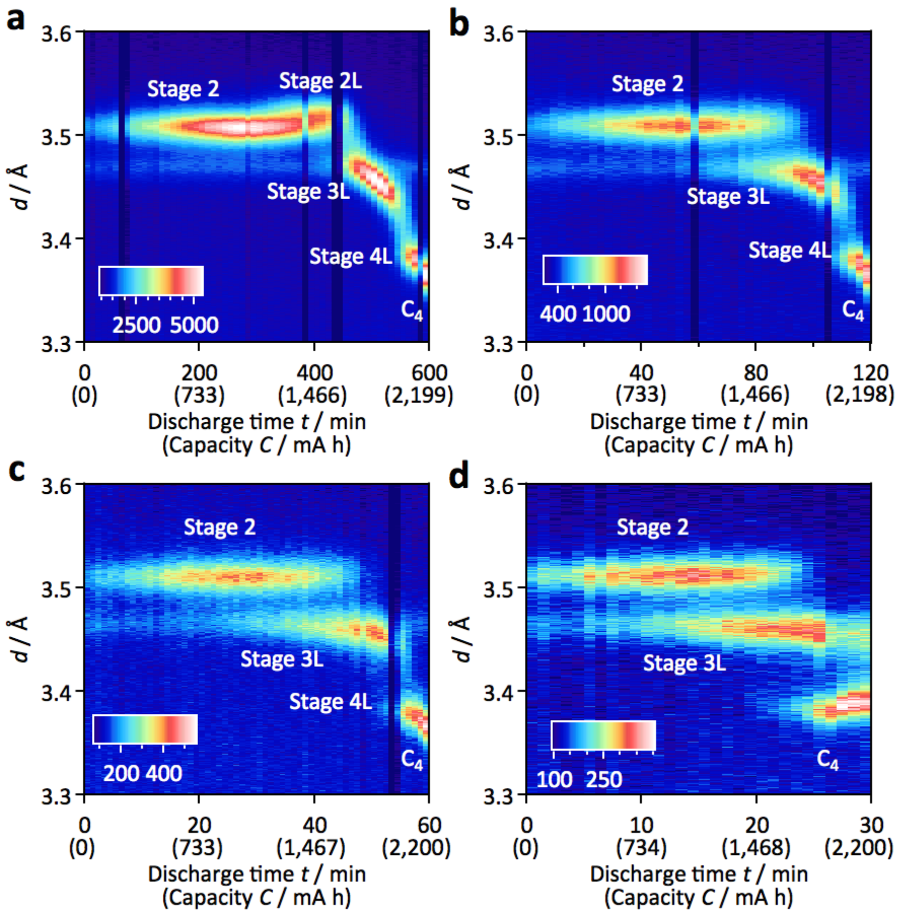

Supplementary Figure S3 | Rate dependence of the phase change mechanism for the carbon material during the discharge reaction. Two-dimensional diffraction profiles near the stage 2, stage 2L, and stage 3L mixture regions at discharge rates of 0.1 (a), 0.5 (b), 1 (c), and 2 C (d). The 2L phase was not observed over the discharge rate of 0.5 C.

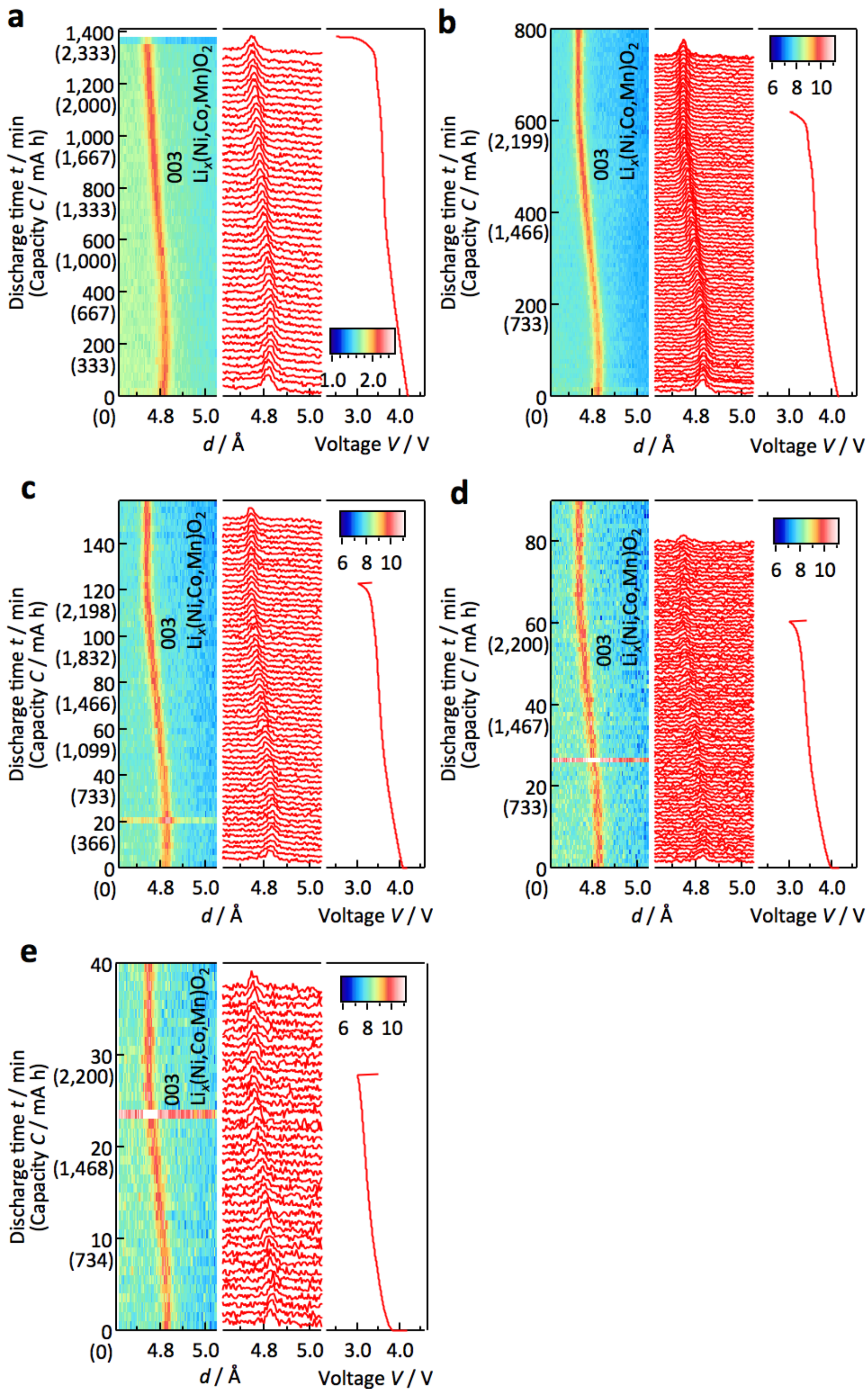

Supplementary Figure S4 | Neutron diffraction profiles of 003 reflections for the cathode material. Graphical and one-dimensional profiles of the diffraction patterns for the cathode during the discharge reactions. The 003 diffraction peaks are indicated in the figure. The discharge rates were 0.05 (a), 0.1 (b), 0.5 (c), 1 (d), and 2 C (e). The voltage profiles of the discharge reactions are indicated for each diffraction profile.

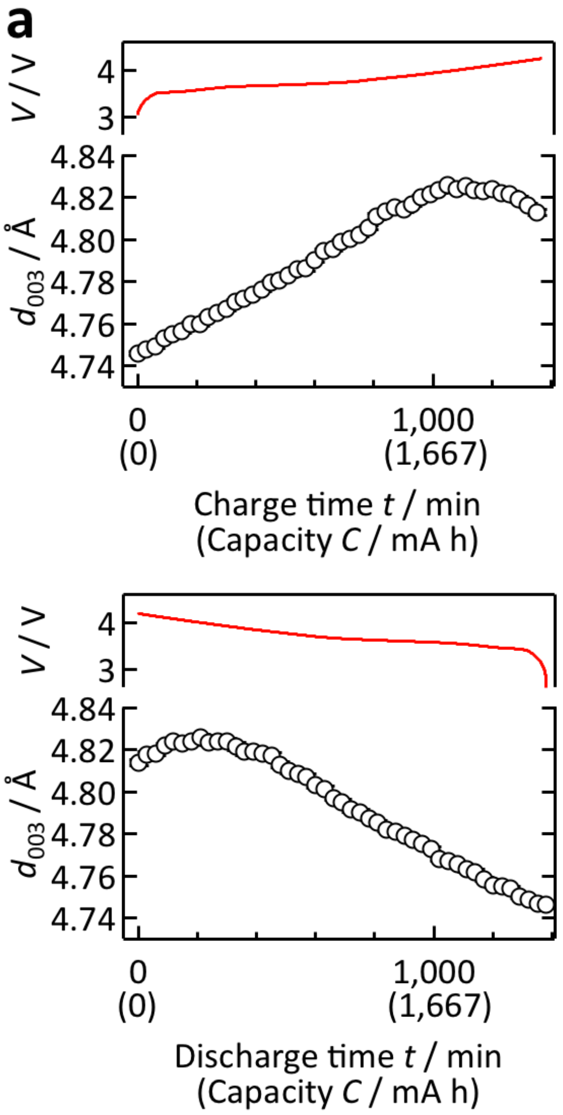

Supplementary Figure S5a

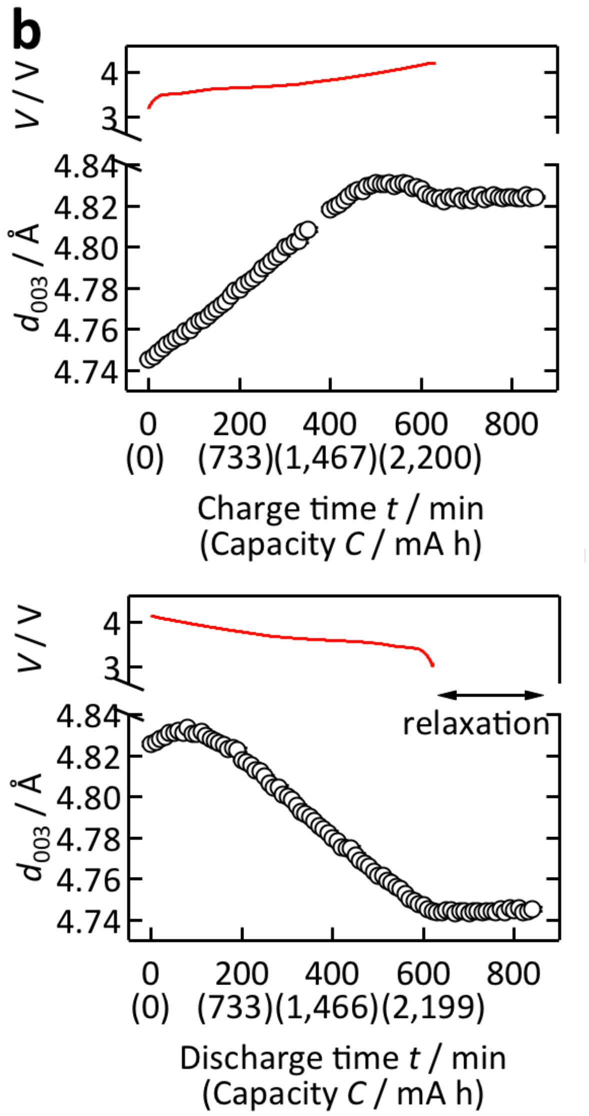

Supplementary Figure S5b

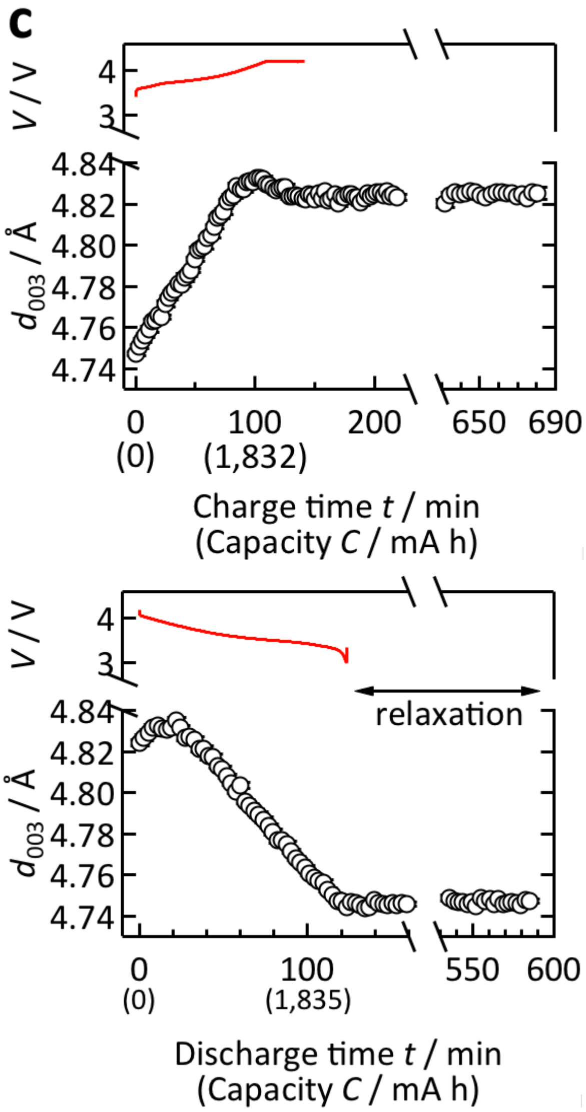

Supplementary Figure S5c

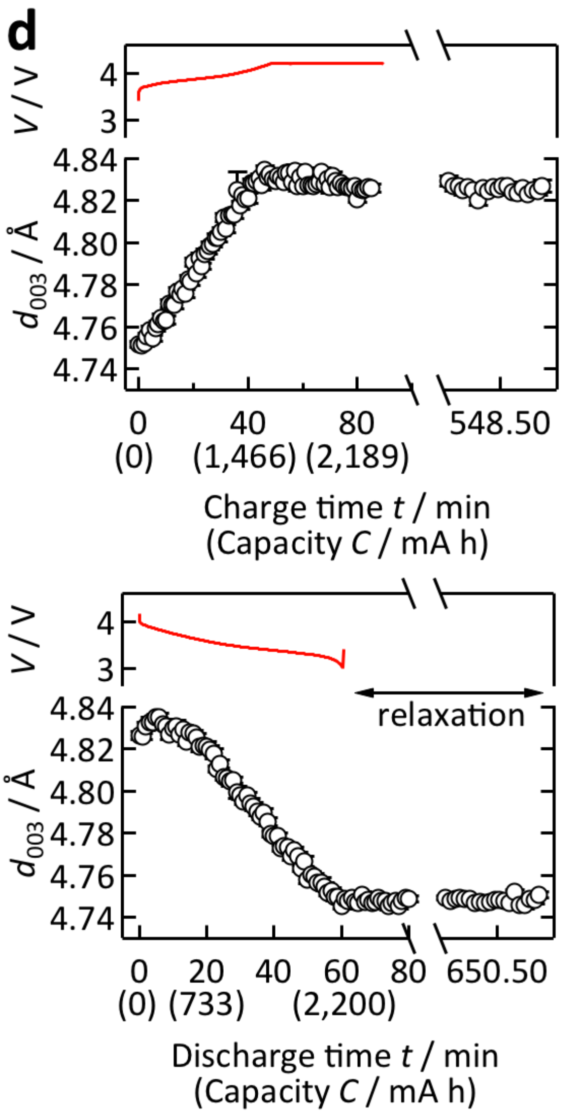

Supplementary Figure S5d

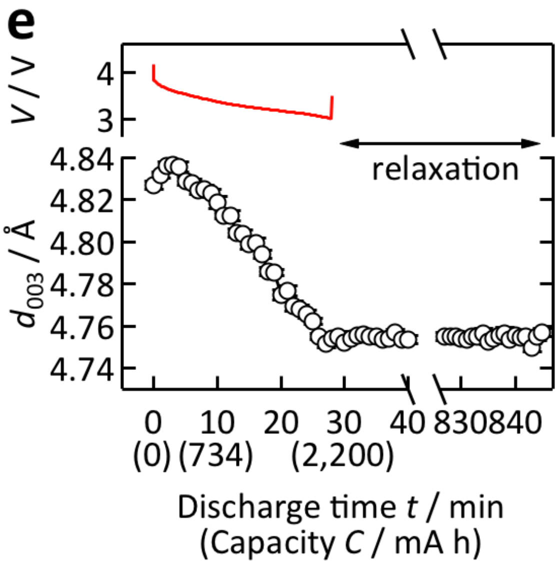

Supplementary Figure S5e

Supplementary Figure S5 | Structural changes during battery reactions. *d*-value changes of the 003 reflection for the cathode $Li(Ni,Mn,Co)O_2$ during the charge and discharge reactions. The charge and discharge rates were 0.05 (a), 0.1 (b), 0.5 (c), 1 (d), and 2 C (e). The profile changes at the 2 C rate are only indicated for the discharge process because the charge reaction was conducted at the 1 C rate. The voltage profiles of the charge and discharge reactions are indicated in each diffraction profile.

Supplementary References

1. Idemoto, Y., Takanashi, Y. & Kitamura, N. Dependence of property, crystal structure and electrode characteristics on heat treatment condition of $Li_{1.041}Ni_{0.8}Co_{0.2}O_y$ as a cathode active material for Li secondary battery. *Electrochemistry* **77**, 945-950 (2009).
2. David, W. I. F. *et al.* Crystal structure and bonding of ordered C_{60}. *Nature* **353**, 147-149 (1991).
3. Straumanis, M. E. & Yu, L. S. Lattice parameters, densities, expansion coefficients and perfection of structure of Cu and of Cu-In α phase. *Acta Crystall. Sect. A* **25**, 676–682 (1969).

4. Otte, H. M. Lattice parameter determinations with an X-ray spectrogoniometer by the Debye-Scherrer method and the effect of specimen condition. *J. Appl. Phys.* **32**, 1536–1546 (1961).

5. Mao, H. K., Bassett, W. A. & Takahashi, T. Effect of pressure on crystal structure and lattice parameters of iron up to 300 kbar. *J. Appl. Phys.* **38**, 272–276 (1967).
